# Supplementary material for: Vigilance state dissociation induced by 5-MeO-DMT in mice
Source: Commun Biol. 2026 Jan 5;9:163. doi: 10.1038/s42003-025-09412-x (PMC12873260; doi:10.1038/s42003-025-09412-x)
Supplement: Supplementary file 2 — Supplementary Information [file 42003_2025_9412_MOESM2_ESM.pdf]

Supplementary figures

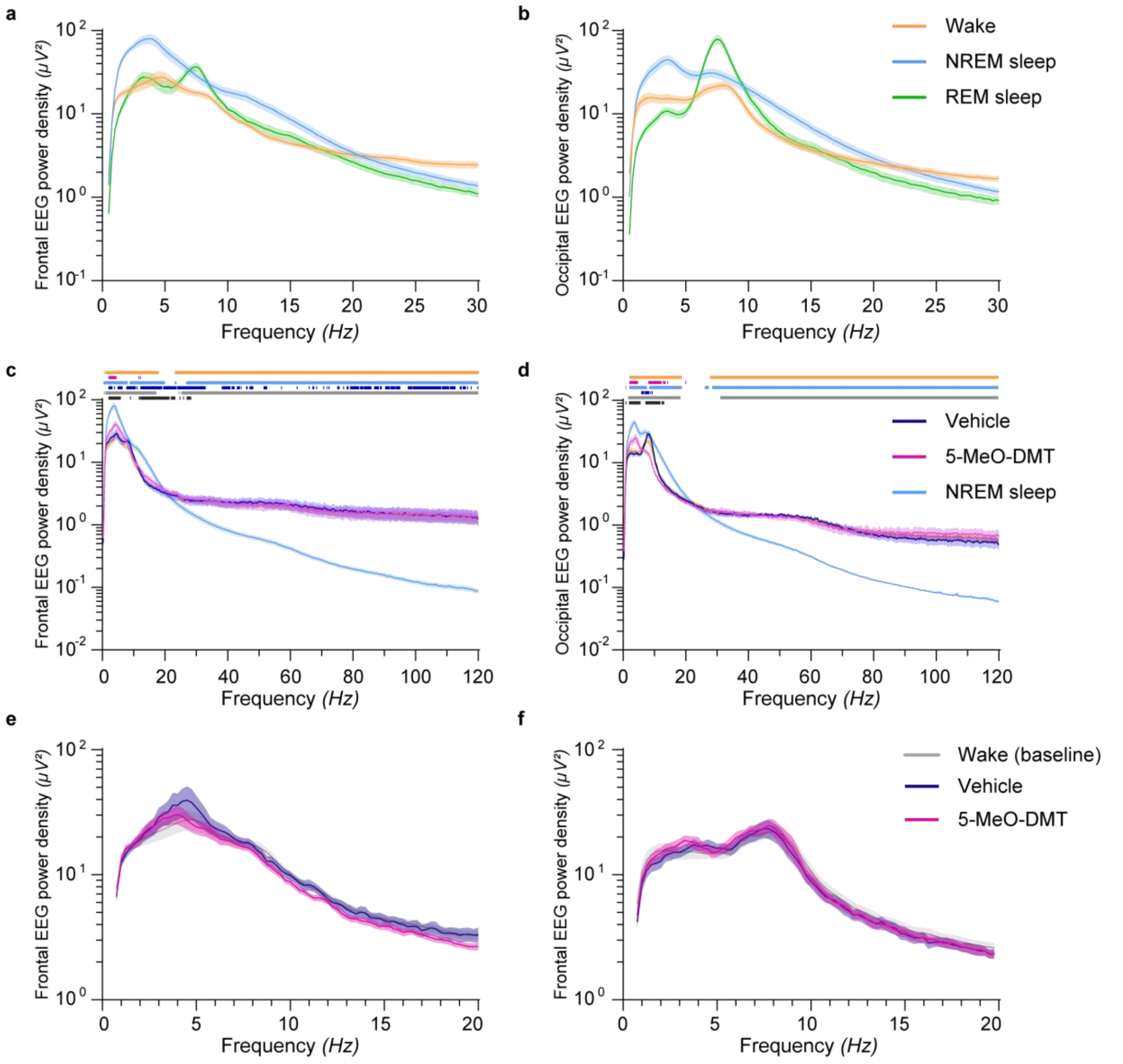

Supplementary Figure 1 | 5-MeO-DMT induced state is characterised by wake-like levels of fast frequencies. a-b. Spectral analysis of frontal (**a**) and occipital (**b**) EEG during wake, NREM sleep and REM sleep (**a.** n = 8; **b.** n = 7). **c-d.** Broader spectral analysis of baseline NREM, wake + vehicle and wake + 5-MeO-DMT in the frontal (**c**) and occipital (**d**) EEG derivations (**c.** n = 8; RM Two-way ANOVA, effect of frequency*condition $F_{1401,9807}$ = 125.9 (GG), p < 0.0001; **d.** n = 7; RM Two-way ANOVA, effect of frequency*condition $F_{1404,8424}$ = 77.03 (GG), p < 0.0001). **e-f.** Spectral analysis of frontal (**e**) and occipital (**f**) EEG during baseline wake, and wake episodes after 5-MeO-DMT or vehicle treatment taken between 1 – 2 hours after the injection (**e.** n = 8; ME analysis, effect of frequency*condition $F_{1.368,9.541}$ = 2.49 (GG), p = 0.14; **f.** n = 7; ME analysis, effect of frequency*condition $F_{2.807,16.77}$ = 1.901 (GG), p

= 0.17). Horizontal line denotes a significant difference between (from bottom to top) vehicle and 5-MeO-DMT (*black*), 5-MeO-DMT and NREM sleep (*grey*), vehicle and wake (*dark blue*), vehicle and NREM sleep (*light blue*), 5-MEO-DMT and wake (pink), wake and NREM sleep (*orange*) (Fisher's LSD post hoc). Mean + SEM.

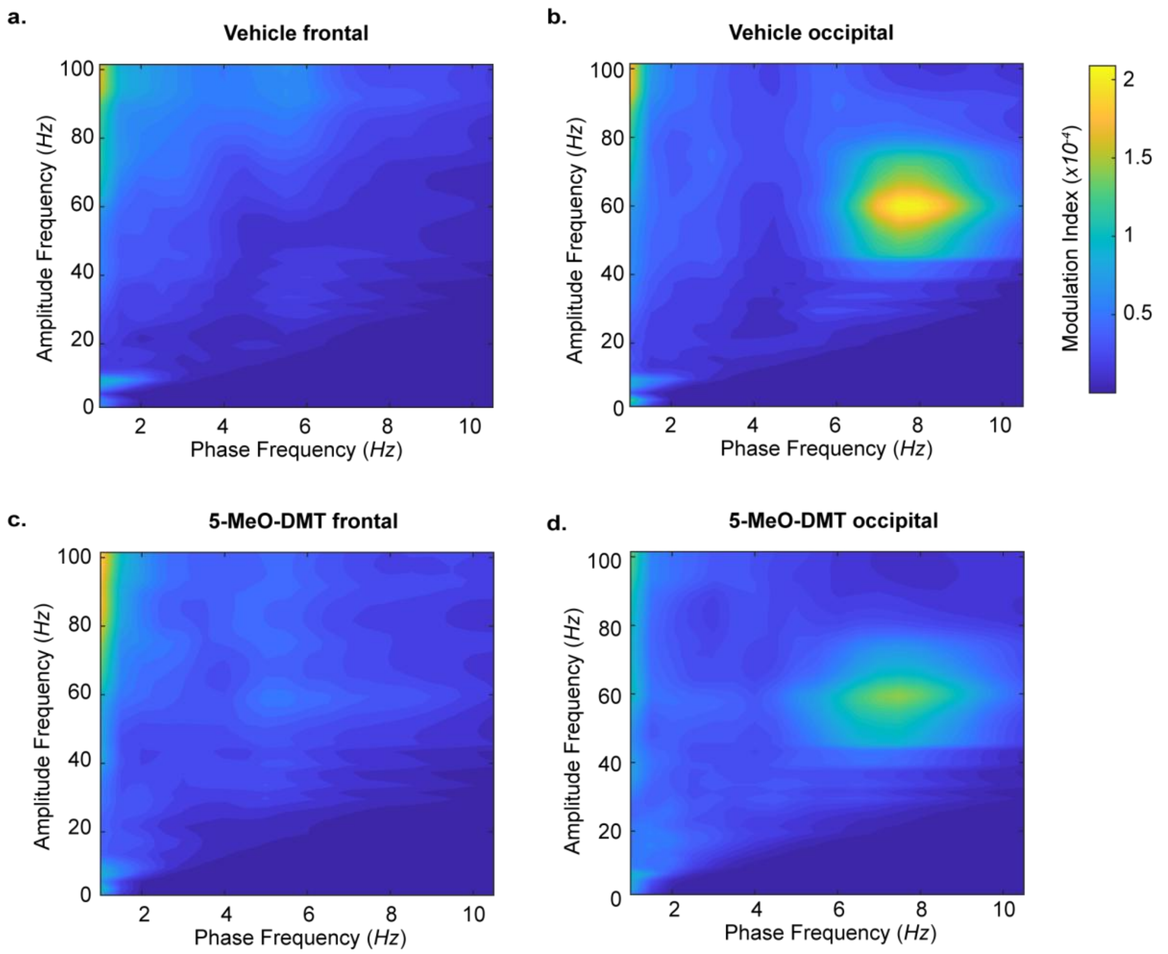

Supplementary Figure 2 | 5-MeO-DMT does not strongly change cross-frequency EEG coupling. a-b. Modulation index calculated in the EEG frontal (**a.**, n = 8) and occipital (**b**, n = 7) in awake mice following an injection of vehicle. **c-d.** Modulation index calculated in the EEG frontal (**c**, n = 8) and occipital (**d**, n = 7) in awake mice following an injection of 5-MeO-DMT.

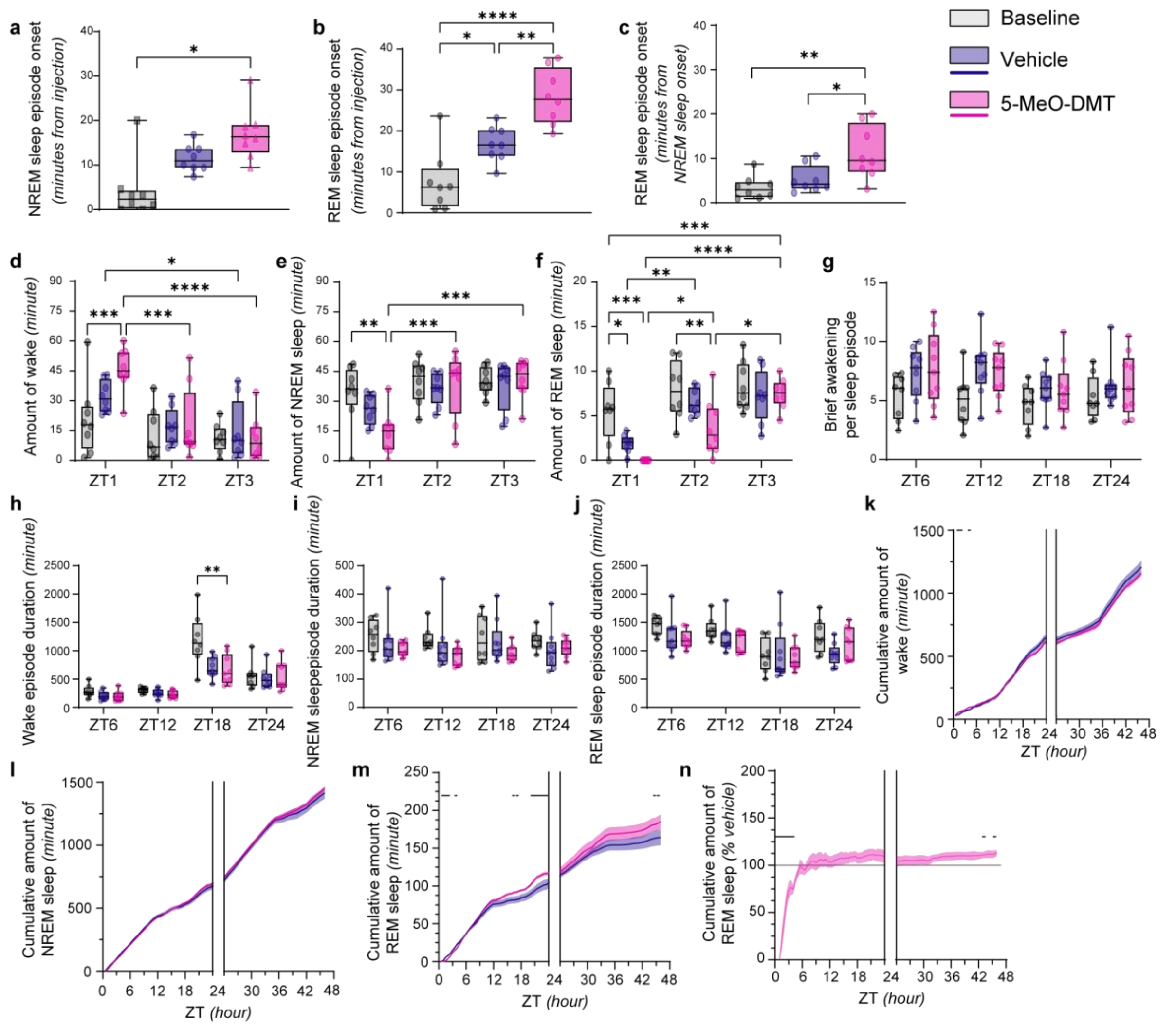

Supplementary Figure 3 | 5-MeO-DMT increases NREM and REM sleep latency and results to a delayed increase in REM sleep. a-b. Latency to NREM sleep onset (**a**) and REM sleep onset (**b**) from the time of injection. (**a.** n = 8; Friedman test $\chi^2(2) = 7.75$, $p < 0.05$; Dunn's $p < 0.05$; **b.** n = 8; RM-one-way ANOVA $F_{2,14} = 25.22$; $p < 0.0001$, Tukey's $p < 0.05$). **c.** REM sleep latency from NREM sleep onset (n = 8 RM-one-way ANOVA, $F_{2,14} = 7.16$; $p < 0.01$, Tukey's $p < 0.05$). **d-f.** Amount of time spent in wake (**d**), NREM sleep (**e**) and REM sleep (**f**) per hour. (**d.** n = 8; ME analysis, effect of quantity*ZT: $F_{1,9} = 210.4$, $p < 0.0001$; **e.** n = 8; ME analysis, effect of quantity*ZT: $F_{1,9} = 159.1$, $p < 0.0001$; **f.** ME analysis, effect of quantity*ZT: $F_{1,9} = 193.9$, $p < 0.0001$;). **g.** Amount of brief awakening per NREM sleep episode (n = 8; ME analysis, effect of condition: $F_{1.75,13.91} = 10.28$ (GG), $p < 0.01$: Tukey's post hoc $p > 0.05$ for baseline against all other conditions). **h-j.** Average wake (**h**), NREM sleep (**i**) and REM sleep (**j**) episode duration (**h.** n = 8; RM two-way ANOVA, effect of condition *ZT: $F_{1.7,10.97} = 7.84$, $p < 0.05$ (GG); Tukey's post hoc; **i.** n = 8; RM two-way ANOVA, effect of condition *ZT: $F_{3.30,20.87} = 0.093$, $p = 0.45$ (GG); **j.** n = 8; RM two-way ANOVA, effect of condition *ZT: $F_{2.56,16.20} = 1.093$ (GG), $p = 0.37$; Effect of ZT $F_{1.66,14.93} = 13.24$ (GG); Tukey's post hoc > 0.05 for ZT18

against all other, and ZT6 against ZT24). **k-m.** Cumulative time spent in wake (**k**), NREM (**l**) and REM (**m-n**) sleep across 48 hours. (**ZT0-ZT24, k.** n = 8; RM 2-way ANOVA, effect of ZT*condition $F_{2.33,16.28}$ = 3.70 (GG), p < 0.05; **l.** n = 8; RM 2-way ANOVA, effect of ZT*condition $F_{2.23,15.61}$ = 2.83 (GG), p = 0.09; **m.** n = 8; RM 2-way ANOVA, effect of ZT*condition $F_{2.63,18.38}$ = 5.90 (GG), p < 0.01; **ZT0-ZT48, k.** n = 4; RM 2-way ANOVA, effect of ZT*condition $F_{1.83,5.49}$ = 3.94 (GG), p = 0.09; **l.** n = 4; RM 2-way ANOVA, effect of ZT*condition $F_{1.73,5.20}$ = 2.25 (GG), p = 0.20; **m.** n = 4; RM 2-way ANOVA, effect of ZT*condition $F_{2.40,7.19}$ = 9.98 (GG), p < 0.01). **n.** Cumulative time spent in REM sleep across 48 hours following an injection of 5-MeO-DNT and normalised to vehicle. (n = 4: ME analysis, effect of ZT*condition $F_{1.68,7.29}$ = 25.56 (GG), p < 0.001; Fisher's LSD post hoc). For all figures, *p<0.05; **p<0.01; ***p<0.001; ****p<0.0001, a black horizontal line denotes a significant difference between 5-MeO-DMT and vehicle conditions, or 100% (**n**). Mean + SEM except boxplots (median + interquartile range).

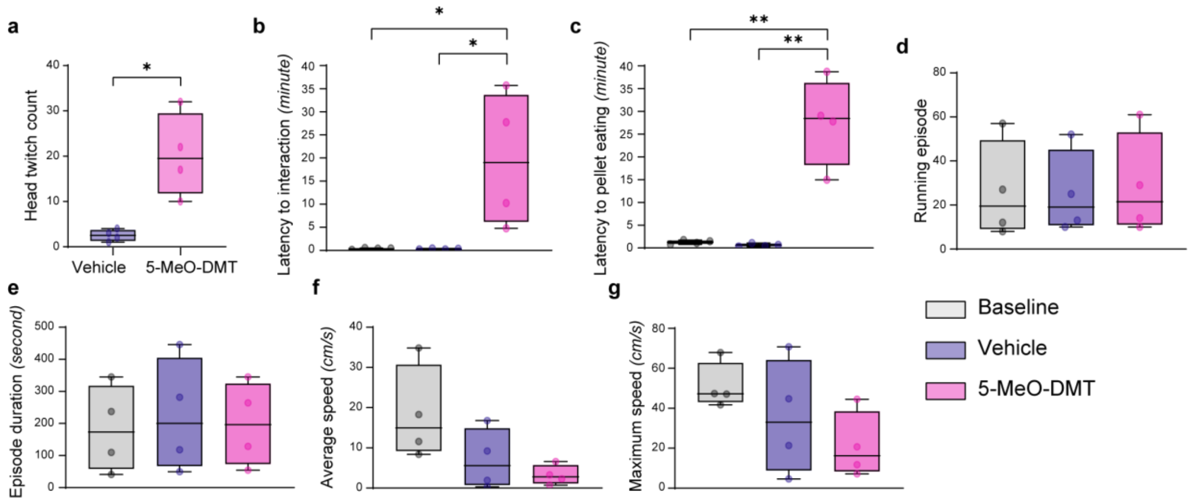

Supplementary Figure 4 | Effects of 5-MeO-DMT on wake behaviour. a. Head twitch response after an injection of 5-MeO-DMT or vehicle (n = 4; t-test, t3 = 3.96; $p < 0.05$). **b.** Latency to bowl interaction (n = 4; RM-one-way ANOVA, $F_{2,6}$ = 7.07 p = 0.026; Tukey's $p < 0.05$). **c.** Latency to sugar pellet eating behaviour (n = 4; RM-one-way ANOVA, $F_{2,6}$ = 28.95 $p < 0.001$; Tukey's $p < 0.01$). **d.** Average amount of running episode within 1 hour of light offset (RM-one-way ANOVA, n = 4; $F_{2,6}$ = 2.854, p = 0.13). **e.** Average running episode duration within one hour of light offset (RM-one-way ANOVA, n = 4; $F_{2,6}$ = 2.16, p = 0.20). **f.** Average running speed of mice within 1 hour of light offset (RM-one-way ANOVA, n = 4; $F_{2,6}$ = 2.81, p = 0.14). **g.** Maximum running speed of mice within 1 hour of light offset (RM-one-way ANOVA, n = 4; $F_{2,6}$ = 1.81; p = 0.24). For all figures, *p<0.05; **p<0.01.Median + interquartile range.

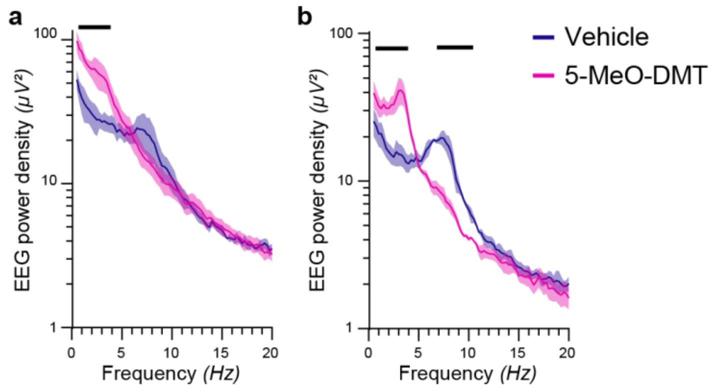

Supplementary Figure 5 | The effects of 5-MeO-DMT on the brain state of animals wearing an oculometer. a-b. Spectral analysis of the frontal (**a**) and occipital (**b**) EEG recordings (**a.** n = 4; ME analysis, effect of frequency*condition, $F_{78,75}$ = 13.22, p < 0.0001; Fisher's LSD p < 0.05; **b.** n = 4; ME analysis, effect of frequency*condition, $F_{78,76}$ = 9.28; p < 0.0001; Fisher's LSD p < 0.05). A black horizontal line denotes a significant difference between vehicle and 5-MeO-DMT for the corresponding frequency. Mean + SEM.

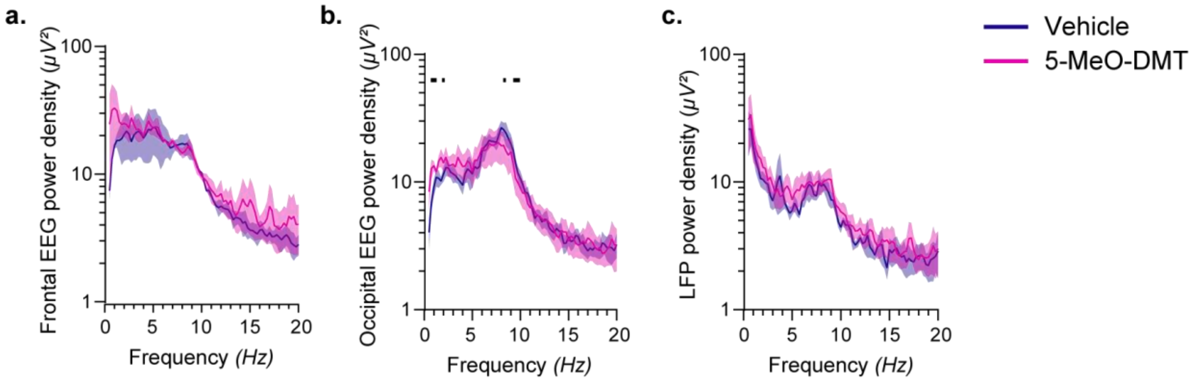

Supplementary Figure 6 | The effects of intracortical injections of 5-MeO-DMT on EEG and LFPs. a-c. Spectral analysis of frontal (**a**) and occipital (**b**) EEG derivations and of the LFP derivation at the level of the local injection (**c**) (**a.** n = 3; ME analysis, effect of frequency*condition: $F_{156,231}$ = 0.93, p = 0.68; **b.** n = 4; RM two-way ANOVA effect of frequency*condition: $F_{156,466}$ = 1.33, p < 0.05; Fisher's LSD p < 0.05; **c.** n = 4; RM two-way ANOVA effect of frequency*condition: $F_{156,468}$ = 0.68, p = 0.99). A black horizontal line denotes a significant difference between vehicle and 5-MeO-DMT for the corresponding frequency. Mean + SEM.

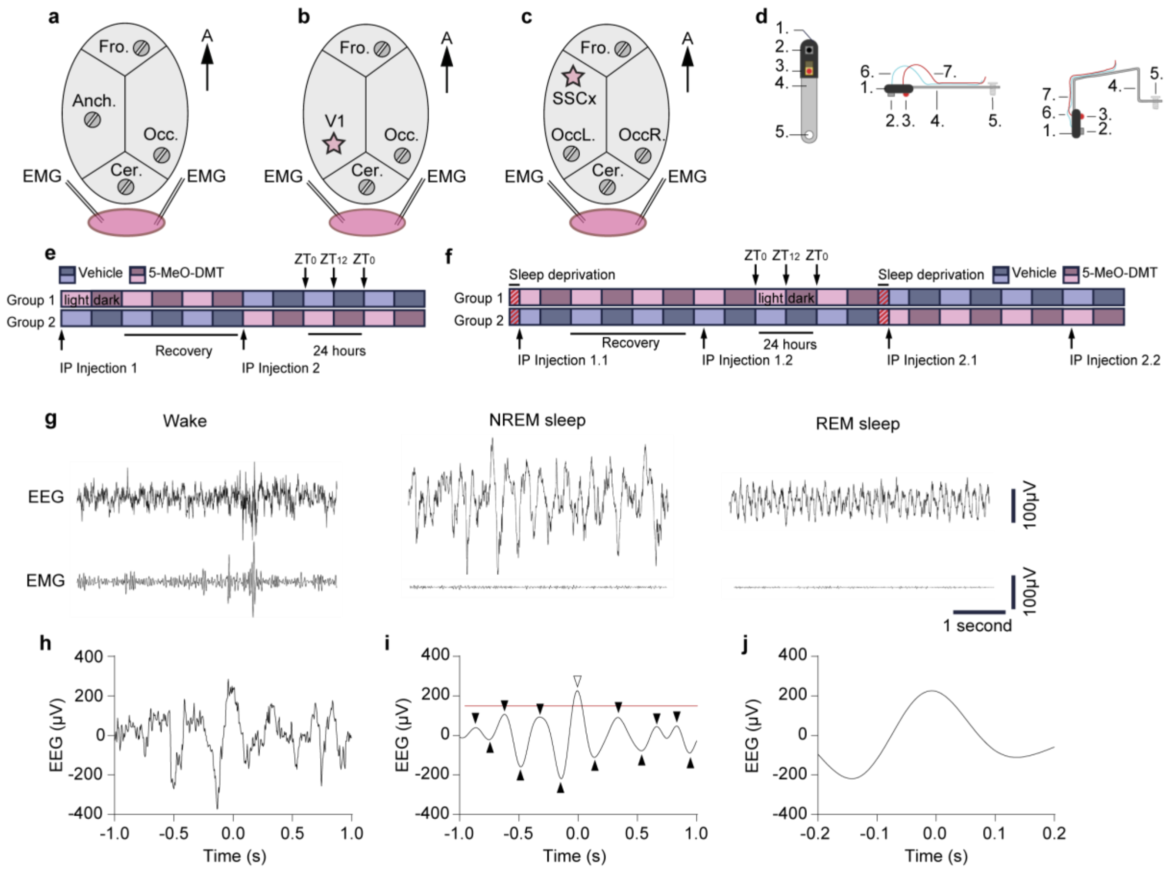

Supplementary Figure 7 | Methods Fro = frontal. Occ = occipital. Cer = cerebellum. Anch = anchor screw. V1 = primary visual cortex. SSCx = somatosensory cortex. **a.** Schematic representation of a mouse skull for EEG implantation surgeries. Fro = frontal. The arrow points towards the anterior part of the animal. The EMG are implanted in the nuchal muscle. **b.** Protocol for counterbalanced, crossover injections. All animals are allocated to one of two groups. Group 1 received 5-MeO-DMT first, group 2 the vehicle. Most animals received their injection at light onset, unless mentioned otherwise. 72 hours after the first injection, the animals received a second injection with the compound they had not yet received. **c.** Sleep deprivation paradigm. The animals are sleep deprived from ZT0 to ZT4 by presenting novel objects. The injections of either 5-MeO-DMT or vehicle are made at ZT4, post-sleep deprivation. Both groups are sleep deprived at the same time. The injections 1.1 and 1.2 are the same substances and the injection 2.1 and 2.2 are the same substances, i.e.: if 1.1 was vehicle, 1.2 was vehicle and 2.1 and 2.2 were 5-MeO-DMT. **d.** Schematic of the oculometer (not to scale) facing view (left), profile view (right) unfolded (top) or folded and ready to be attached (bottom). 1. Sugru paste. 2. Camera. 3. LED. 4. Aluminium plate. 5. Screw. 6. Optic fibre. 7. Insulated copper cable. **e.** Representative EEG and EMG signals for all vigilance states. Wake is associated with EEG signals of fast frequency and low amplitude with high EMG activity. NREM sleep is defined by large slow-waves of high amplitude in the frontal part of the brain, with low-to-absent muscle activity. In REM sleep, the occipital EEG shows

an increased theta activity due to the proximity of the electrode to the hippocampus and a flat EMG due to muscle atonia. **f.** Schematic representation of a mouse skull for EEG implantation surgeries with laminar probe. **g.** Schematic representation of a mouse skull for EEG and cannula implantation surgeries. **h-j.** Representative example of the slow wave detection. First the raw signal (**h**) is filtered (**i**). Then, all local maxima and minima are detected and processed (black arrows). For the multi-unit analysis, only the waves with an amplitude above the red threshold are kept (white arrow), shown in detail in **j**.

a.

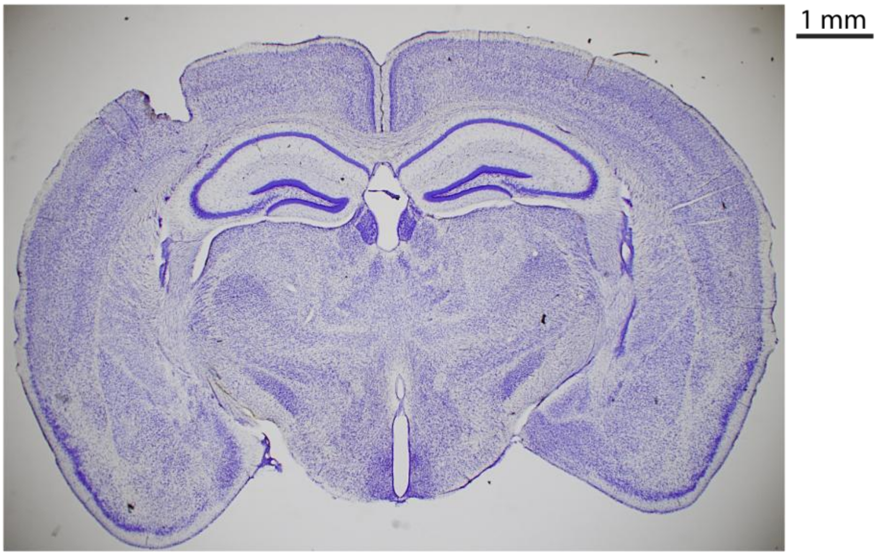

Supplementary Figure 8 | Histology a. Representative histology picture in an animal implanted with a cannula stained with cresyl violet, located at -1.9 antero-posterior. The cannula track is located at the junction of the barrel field in the primary somatosensory cortex and the posterior parietal cortex.

Frontal	Slope (*x10^-3*)	Std. error (*x10^-5*)	R^2
**Baseline - Wake**	-3.78	3.89	0.96
**Baseline - NREM**	-14.33	13.74	0.96
**Vehicle**	-4.07	4.90	0.95
**5-MeO-DMT**	-4.18	6.48	0.91

Occipital	Slope (*x10^-3*)	Std. error (*x10^-5*)	R^2
**Baseline - Wake**	-6.26	7.17	0.95
**Baseline - NREM**	-15.57	15.91	0.96
**Vehicle**	-6.40	9.17	0.92
**5-MeO-DMT**	-5.97	8.87	0.91

Supplementary Table 1 | Spectral slopes. Spectral slopes calculated between 20–120 Hz (in linear–log space) for baseline wakefulness and NREM sleep, as well as wakefulness following vehicle and 5-MeO-DMT injections, with the corresponding standard error and goodness of fit (R^2). There was a significant effect of vigilance state on the EEG spectral slope (Frontal: $F_{3,1596} = 3951$, $p < 0.0001$; Occipital: $F_{3,1596} = 1878$, $p < 0.0001$).
