# Supplementary material for: Vigilance state dissociation induced by 5-MeO-DMT in mice
Source: Commun Biol. 2026 Jan 5;9:163. doi: 10.1038/s42003-025-09412-x (PMC12873260; doi:10.1038/s42003-025-09412-x)
Supplement: Supplementary file 3 — Description of Additional Supplementary Files [file 42003_2025_9412_MOESM3_ESM.pdf]

Description of Additional Supplementary Files

File name: Supplementary Movie 1

Description: Waking behaviour in mice following the injection of vehicle or 5-MeODMT

File name: Supplementary Movie 2

Description: Mouse interacting with a bowl after an injection of vehicle or 5-MeO-DMT

File name: Supplementary Movie 3

Description: Mouse interacting with a running wheel after an injection of vehicle or 5-MeO-DMT

File name: Supplementary Movie 4

Description: Representative EEG and LFP signals of a mouse in NREM sleep, REM sleep, wake with vehicle and wake with 5-MeO-DMT

File name: Supplementary Movie 5

Description: Mice wearing the oculometer with and without 5-MeO-DMT

File name: Supplementary Movie 6

Description: Oculometer recording technique

File name: Supplementary Movie 7

Description: 5-MeO-DMT transiently increases pupil diameter. The two graphs at the top are visual representation of the pupil size after vehicle (left, grey) and 5-MeO-DMT (right, red) injections against baseline (full black circle) based on the averaged pupil size over 60-minutes following an injection (bottom).
